# Testicular Implant Complications after Transmasculine Gender Affirming Surgery

**DOI:** 10.1590/S1677-5538.IBJU.2024.0427

**Published:** 2025-01-13

**Authors:** Patrick Ho, Emily Schmidt-Beuchat, Michaela Sljivich, Miroslav Djordjevic, Ethan Nyein, Rajveer S. Purohit

**Affiliations:** 1 Mount Sinai Hospital Department of Urology New York USA Department of Urology, Mount Sinai Hospital, New York, USA; 2 Mount Sinai Hospital Department of Obstetrics and Gynecology New York USA Department of Obstetrics and Gynecology, Mount Sinai Hospital, New York, USA; 3 University of Belgrade Department of Surgery and Urology Belgrade Serbia Department of Surgery and Urology, University of Belgrade, Belgrade, Serbia

**Keywords:** Surgical Procedures, Operative, Transgender Persons, complications [Subheading]

## Abstract

**Purpose:**

Complications from testicular implantation in transgender men can cause significant distress, repeat visits to the emergency department, and require reoperation for explantation. Outcomes for these implants have not been well described in the literature. This study compares patient and surgery specific factors with complications from testicular implants in transgender men.

**Materials and Methods:**

We performed a retrospective review of patients who underwent testicular implantation. Surgery was standardized across patients with placement through incisions at the top of the labia majora or medially during metoidioplasty. Complication rates, including infection, erosion, migration, and pain requiring removal was compared with patient factors, including body mass index (BMI), smoking status, and implant size.

**Results:**

Of the 116 testicular implants, 12% had a complication requiring removal. The most common reason for removal was erosion of the prosthesis, which occurred in 6 instances. Migration was a relatively frequent complaint, with 10% of patients noting relocation of an implant. However, only 4 implants ultimately underwent reoperation for migration. Four implants caused enough pain to require reoperation. On logistic regression of BMI, age, smoking status, and immunocompromised state on removal of prosthesis, no factor was found to be a significant predictor of removal. Increasing implant size was not associated with an increased likelihood of removal.

**Conclusions:**

Complications after testicular implants in transgender men are not uncommon events. Although there appears to be a growing trend toward smaller prostheses in the literature, our data suggest that implant size is not a significant predictor of complications requiring prosthetic removal.

## INTRODUCTION

Testicular prostheses have been used since the 1940s for the variety of etiologies that cause a testicle to be absent, such as castration for prostate cancer, after testicular torsion, undescended testicles, or orchiectomy for testicular cancer (1, 2).

The complications of testicular prostheses in cis-gendered men have been well documented and include extrusion, pain, and infection. Testicular prostheses in the modern era with saline-filled implants have been reported to be safe and well-tolerated in cisgender adult and pediatric patients (3, 4). The removal rate of testicular prostheses placed after radical orchiectomy for testicular cancer has been reported to be <0.5% (5).

A variety of techniques have been described for scrotoplasty with masculinizing gender affirming surgery including those without testicular implants or placed in a staged fashion such as the Ghent technique (6) or scrotoplasty with concomitant testicular implants. The complication rates of testicular prostheses for gender affirming surgery are not well studied. There is a dearth of revision and explantation rates in transgender men who have had implantation of testicular prostheses (7). Placement of testicular implants in transgender men is potentially different from cisgender men for a variety of hypothetical reasons including differences between labial and scrotal sizes, potential differences in skin thickness and fat distribution. Furthermore, transgender men can often be undergoing a significantly larger surgery at the time of implant placement (metoidioplasty with or without hysterectomy (8)) compared to cis-gender men (orchiectomy). Different factors have been proposed to contribute to prosthetic complications, including smoking, surgical technique, and implant size (9, 10). We hypothesized that the rate of complications in testicular implants would be higher in transgender men compared to that of cisgender men, and that larger implant size would be associated with an increased complication rate. The purpose of this study is to identify the risk of removal of testicular implants in transgender men and factors that contribute to complications.

## MATERIALS AND METHODS

A retrospective review was performed of patients who underwent transmasculine gender affirming surgery from 2021 to 2023 at a single institution, as part of an IRB-approved study (IRB 20-01505). Patients were included if their surgery was a metoidioplasty with scrotoplasty and insertion of testicular prostheses. Patients were excluded from analysis if their surgery was not their index surgery or if data of interest were omitted on record review, such as implant size.

**Figure 1:**
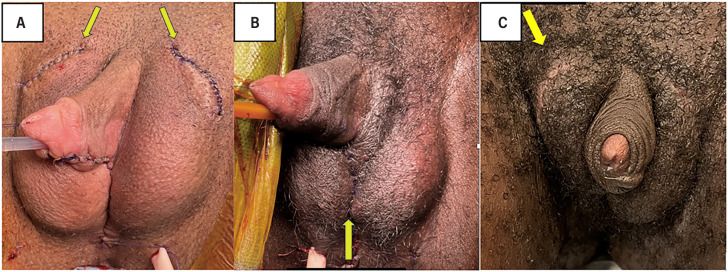
Approaches to Testicular Prosthesis Implantation.

Two senior surgeons performed all testicular prosthesis implantations. Implantation was standardized across all patients with one of two techniques, with Coloplast® Torosa silicone prostheses placed in pockets created by incisions at the top of the labia majora or blunt dissection of the labia majora medially during metoidioplasty. Incisions at the top of the labia majora, labeled a superolateral approach, create dartos pockets in the newly formed scrotum for the implants. The implants are placed superficial to the Martius fat pad and have not typically been anchored in place with a suture (Figure-1A). In the medial approach, the labia majora are dissected and joined in the midline to create the scrotum. Each side is then opened bluntly on the medial aspect to create pockets for the implants. These implants are also placed superficial to the labial fat pads. Medial insertion of implants avoids the need for additional incisions and minimizes scar (Figure-1B).

A) Superolateral approach: Incisions are made at the superolateral aspects of the labia majora to create dartos pockets for the silicone testicular implants; B) Medial approach: Pockets for the silicone implant are created at the medial aspects of the labia majora; C) Example of migrated right testicular prosthesis.

Demographic variables were collected for each patient, including age, body mass index (BMI), and current or former smoking status. The presence of any comorbid immunocompromising disease, including diabetes, HIV infection, or chronic steroid use, was also measured. The primary outcome of interest was a post-operative complication, such as infection, erosion of the prostheses, implant migration, or pain, that required implant removal. Implant removal was compared with patient factors, including age, body mass index (BMI), smoking status, implant size, and immunocompromised state. For the purposes of analyzing implant removal, each implant was considered an observation since not all patients who underwent removal had bilateral explantation.

Statistics were performed using Stata 16 (StataCorp, College Station, TX). Pearson's chi-squared test was performed to evaluate differences in rates of implant removal between implant technique. Logistic regression was performed to identify patient factors associated with complications requiring implant removal. Statistical test results were deemed significant for p-values less than 0.05. Institutional Review Board approval for this observational study was obtained through our institution's Program for the Protection of Human Subjects.

## RESULTS

Fifty-eight patients who underwent scrotoplasty with bilateral testicular prostheses insertion met the inclusion criteria for this study. The median follow-up period was 28 weeks, and the median patient age was 30 years old. Nearly 75% of study participants had a BMI < 30. Twenty-six patients (45%) were current or former smokers, and three patients had a comorbid immunocompromising condition. Forty-seven patients (81%) had testicular prosthesis placement via the superior-lateral approach.

Of the different complications, migration (Figure-1C) was the most frequent complaint noted in postoperative visits, with 10% of patients noting relocation of one or more of their prostheses postoperatively. However, only 4 implants (3%) ultimately underwent reoperation for migration. Five patients experienced prosthesis erosion requiring removal, while two others had implant-related pain that also required removal. One patient developed cellulitis overlying their implants, which was managed conservatively with antibiotics. The median time to complication was 22 days postoperatively.

Of the 116 testicular implants, 14 implants (12%) had a complication that required removal. The most common reason for post-operative removal was erosion of the prosthesis, which occurred in 6 instances (5%). Eroded implants were removed in the clinic or emergency department. They required either aspiration or manipulation out of skin opening followed by packing. Four implants (3%) caused significant enough pain to require reoperation for removal. By technique, 1 of 22 (5%) implants by medial approach underwent removal compared to 13 of 94 (14%) implants by superior-lateral approach (p=0.23).

The rate of implant removal was compared against patient factors (Table-1). On univariable logistic regression of BMI, age, smoking status, and immunocompromised state on post-operative removal of prosthesis, no factor was found to be a significant predictor of subsequent removal. Furthermore, increasing implant size was not associated with an increased odds ratio of prosthetic removal.

**Table 1:** Implants Removed by Patient-specific and Surgical Factors.

		Implants Removed (%)	Total Implants
**Age (Years)**			
	20-29	6 (10)	58
	30-39	0 (0)	28
	40-49	8 (33)	24
	50-59	0 (0)	6
**BMI**			
	<18.5	0 (0)	2
	18.5 - 24	3 (8)	36
	25 - 29	5 (11)	46
	30 - 39	6 (20)	30
	>= 40	0 (0)	2
**Smoker**			
	Yes	9 (14)	64
	No	5 (10)	52
**Immunocompromised**			
	Yes	2 (33)	6
	No	12 (11)	110
	Implant Size		
	Small	7 (18)	40
	Medium	3 (7)	44
	Large	4 (13)	32
**Prosthetic Technique**			
	Superolateral	13 (14)	94
	Medial	1 (5)	22
**Univariate Logistic Regression on Post-operative removal**			
		Odds Ratio (95% (CI)	p value
Age		1.03 (0.96 - 1.11)	0.41
BMI		1.01 (0.88 - 1.16)	0.32
**Implant Size**			
	Small (referent)	-	-
	Medium	0.80 (0.14 - 4.51)	0.80
	Large	1.23 (0.21 - 7.15)	0.82
**Current or Former Smoker**		0.98 (0.23 - 4.10)	0.98

## DISCUSSION

Rates of testicular prosthesis complications from transgender surgery described in a review by Fascelli et al. are wide-ranging, including infection rates of 3-11% and extrusion rates of 7-14% (7). These rates are subject to overlapping etiologies, however, such as an infection causing wound breakdown and ultimately implant extrusion. Therefore, in our study, we focused on rates of post-operative removal to compare against potential risk factors.

A study of 206 patients who underwent scrotoplasty and testicular implants from Amsterdam University Medical Center found an explantation rate of 13% for their prostheses (9). At this center, implants were increasingly placed during a second stage surgery. Prior studies have also suggested a delayed prosthetic implantation approach to gender affirming surgery of at least six months after the index procedure (6, 7, 10-12). In our study patients underwent a one-stage metoidioplasty surgery, which includes lengthening of the clitoris and urethra along with scrotoplasty with testicular prosthesis implantation as described by Djordjevic et al. (13). However, the comparable rate of explantation (12%) in our one-stage cohort suggest that, at least for testicular prostheses, immediate implantation is possible.

There was a slightly higher rate of explants for patients who were current or former smokers (14% vs. 10%), however the likelihood of post-operative removal was not increased by smoking status on regression analysis. This is in contrast to the study from Amsterdam University that found smoking to be a significant risk factor for infection. The idea that poor wound healing could contribute to higher rates of implant removal led us to examine rates of comorbid immunocompromising conditions, such as diabetes, HIV infection, or chronic steroid use, in patients requiring removal. If a significant risk factor, strategies such as lowering HgA1c or delaying prosthetic implantation after metoidioplasty, may be advisable. Only 3 patients in our data set had an immunocompromising condition, with one of them requiring explantation of both prostheses. This higher rate of explantation requires future examination of a larger sample of immunocompromised patients.

We found that migration was the most common complication after testicular implant placement. This complication is significant for altering the appearance of the scrotum but can also interfere with urination and directing the urinary stream. During the study period we did not routinely suture the implant in place as we had found, anecdotally, prior to the study period that implant migration occurred despite the placement of anchoring sutures and these sutures can distort the appearance of the scrotum.

Given that one of the most common complications is prosthesis extrusion, technique must be given careful consideration. Our study documented two techniques for implant pouch creation. There was not a significant difference between explantation rate on chi-squared analysis; however, the vast majority of implants were performed via the superior-lateral approach. Going forward, surgical techniques can be compared and trialed against each other to minimize erosion rates. Kang et al. emphasizes the importance of minimizing skin tension for the prosthesis pouch to prevent erosion (12). They cite an example of pouch formation in the scrotal reconstruction of a patient who suffered scrotal trauma; surgeons used Foley catheter balloons as tissue expanders in the perineal-scrotal region to create new pockets for the native testes (14). Postoperative care is yet another area of study that can improve rates of complications and prosthesis explantation. In our institution, all patients after testicular implants are given the same post-operative instructions to avoid sitting, heavy lifting, and walking more than 200 steps per day.

In addition to understanding complication rates and their risk factors, another future area of research is patient satisfaction with testicular implants. Patients who have had testicular prostheses implanted after surgical castration for prostate cancer report greater satisfaction compared to orchiectomy alone (15). The complication rate for transgender men is much higher than that of cisgender men and can cause significant distress. It would be important to understand the level of patient satisfaction for various prosthesis factors, such as size, positioning, and comfort, so that we can weigh these against the costs and risks of implantation.

A major limitation of our study is the duration of follow-up, with a median follow-up of just over 6 months. This poses the problem of underestimating the complication rate, if patients were to seek care outside of our institution or experience complications going forward. We also only have one type of silicone implant available at our center, which limits the comparison of different implant types on complication rates. The retrospective nature of our study is another limitation of the data to predict an implant complication based on risk factors. For example, if surgical technique was chosen based on a perceived likelihood of complication, it loses its predictive power in a regression analysis.

Testicular prostheses have become increasingly used for gender affirming surgery. The growth of their use in the transgender population requires increased attention to complication rates, which thus far have been reported to be at least twenty-fold greater than in cisgender men.

In our series, the complication rate of testicular implants requiring removal was 12%. Our study contributes to the existing literature by showing that single-stage testicular implantation during metoidioplasty carries the same rate of postoperative removal compared to a staged approach. We also showed that the size of implants did not correlate to complication rate, suggesting that placement of the largest size implant that labial size and cosmetic appearance permits is reasonable. Given our sample size, we could not evaluate medial versus superior-lateral placement of implants. Further prospective study is needed to understand patients at higher risk of implant complications to preempt them during an already long and arduous process of transition.
